# Study on size effect of limestone-concrete composite with different joint inclination angles under uniaxial compression based on the discrete element method

**DOI:** 10.1371/journal.pone.0345367

**Published:** 2026-03-25

**Authors:** Hang Liu, Mingxuan Shen, Bin Du, Jie Wang, Haiyang Chen

**Affiliations:** College of Civil Engineering, Guizhou University, Guiyang, Guizhou, China; China Construction Fourth Engineering Division Corp. Ltd, CHINA

## Abstract

This study utilized synthetic rock mass technology based on the discrete element method implemented in PFC to develop multi-scale limestone-concrete composite (LCC) models in order to examine the size effect of jointed rock-concrete composites (RCC) and assess the impact of joint distribution characteristics on the composite. The variation patterns of the strength properties, deformation characteristics, and failure features with size were analyzed using uniaxial compression tests. Additionally, the impact of the joint dip angle on the size effect of the composite was explored by altering the dip angle range. The results demonstrate that both the uniaxial compressive strength (UCS) and compressive modulus of the LCC exhibit a significant size effect. The calculated difference ratios revealed that the UCS stabilized at dimensions of 600 mm × 1200 mm, whereas the compression modulus decreases by less than 10% and gradually stabilizes. Overall, the deformation characteristics of the composite are less sensitive to size variations than its strength characteristics. During failure, the energy release in the LCC became more dispersed with increasing dimensions. The failure mode transitions from global failure to dispersed localized failure, with crack propagation and stress transfer becoming more dispersed and complex. The range of joint dip angles significantly influences the size effect of the composite. Calculations of the corresponding Δ values indicated that the size effects on UCS and compressive modulus were most pronounced at joint dip angles of 75°–80° and 30°–35°, respectively. Overall, variations in the dip angle range exerted a greater impact on the size effect of UCS than on that of the compressive modulus. These findings provide valuable reference for subsequent research on RCC and related engineering projects.

## 1. Introduction

Concrete, rock, and other quasi-brittle materials exhibit a size effect, specifically manifested as significant variations in strength and deformation characteristics with changes in dimensions. For most civil engineering problems, laboratory studies cannot replicate the actual project scale. Therefore, investigating the size effect in different materials and structures plays a crucial role in applying experimental results to guide engineering practices. RCC is commonly encountered in engineering projects in which concrete structures interface with rock masses to jointly bear loads. Its mechanical properties determine the stability of concrete structures built on rock foundations. However, research on the size effect of RCC remains scarce, as it is a combination of two quasi-brittle materials. The manifestation of the size effect in composite structures and its influencing factors remain unclear. Therefore, investigating the size effect of RCC is essential to facilitate the transfer of experimental research findings into engineering practices.

The size effect is a long-standing problem. Numerous scholars have discovered that the strengths of quasi-brittle materials, such as rocks and concrete, decrease with increasing size and gradually stabilize. Some scholars have offered explanations from different perspectives. Based on statistical analysis, Weibull attributed the size effect to an increase in internal defects within materials as the size increases [1,2]. Bažant interpreted the size effect from the perspective of fracture mechanics, proposing that it arises from the dissipation of strain energy during macrocrack propagation in quasi-brittle materials [3–5]. Hu proposed the boundary effect theory, suggesting that size effect arise from the interaction between the crack propagation zone and the specimen boundary [6,7]. With numerous scholars validating and refining the above theories [8–11], the mechanism of the size effect has become relatively clear. Current research tends to focus more on investigating the influence of different operating conditions or other factors on the size effect [12–16]. The internal composition of concrete is essential for examining the size effect. Wang et al. [17] employed Monte Carlo simulation method to investigate the tensile size effect in concrete. While validating the Weibull size effect formula, they emphasized that the influence of aggregates and voids on the size effect cannot be overlooked. Alam et al. [18] further investigated the internal heterogeneity dimensions and found that the concrete size effect is a function of both structural and heterogeneous dimensions. Similarly, the focus of rock size effect lies in internal defects, such as joints. Through numerical simulations, Zhao et al. [19] investigated the influence of the joint dip angle and size on the rock mass size effect and anisotropy, finding that the rock mechanical properties were most stable when the joint dip angle was 90°. In addition to internal factors, the influence of external conditions warrants consideration. Through Brazilian splitting tests, Su et al. [20] discovered that high temperatures significantly affect the size effect on the tensile strength of red sandstone, with the size effect being least pronounced at 400°C. In their study on the impact of freeze-thaw cycles, Guan et al. [21] discovered that with an increase in the number of these cycles, the size reduction effect in wastewater concrete significantly diminished, and even a size increase effect emerged. Most scholars currently focus on investigating the size effect under multiple factors and conditions, yet pay little attention to the size effect behavior of key engineering structures such as RCC. Therefore, conducting research on the size effect of RCC under random joints has significant theoretical and engineering implications for fundamental infrastructure construction and disaster prevention.

As an effective tool for studying the size effect, numerical simulation methods not only overcome the accuracy limitations of analytical approaches but also address the constraints of experimental research, such as size limitations and high costs. Among these, the discrete element method (DEM), a numerical technique that simulates system behavior by modeling numerous discrete particles and blocks, is widely applied in the modeling of discontinuous rock masses and heterogeneous materials [22–25]. In the domain of jointed rock mass simulation, Ivars et al. [26] introduced a synthetic rock mass (SRM) methodology utilizing the discrete element software Particle Flow Code (PFC). Building on a particle model, this method employs a smooth joint contact model (SJCM) to simulate joint networks. Beyond the mechanical behavior, it also captures the rock mass size effect and anisotropy. Building upon this foundation, numerous scholars have refined SRM technology by integrating various numerical software and structural surface network identification techniques, thereby providing more accurate methods for simulating real engineering rock mass [27–32]. Compared with continuous deformation methods, such as the finite element method (FEM), the DEM is more appropriate for simulating large deformations and crack propagation in geotechnical materials. The SRM technique also addresses the computational complexity associated with large-scale models. Therefore, employing PFC combined with SRM technology to simulate jointed RCC offers distinct advantages over other methods in terms of mechanical behavior, size effect, and fracture processes, representing a more precise solution to the problem.

To conclude, the size effect of RCC is vital in the construction of foundation engineering structures and disaster prevention. Therefore, this study employed PFC^2D^ to establish LCC models at different scales. Using SRM technology, random fractures with varying dip angles were embedded within the limestone model. Uniaxial compression simulations were conducted on the composite models, and comparative analyses were performed to examine the differences in strength characteristics, deformation behavior, and failure patterns across composite models of varying sizes. This study aims to explore the size effect patterns of jointed LCC with different mechanical properties. In addition, by varying the dip angle range of the randomly distributed joints in the limestone, the influence of the joint dip angle on the size effect of the composite material was further analyzed.

## 2. The theory of equivalent rock mass

### 2.1. Synthetic rock mass technology

**Fig 1:**
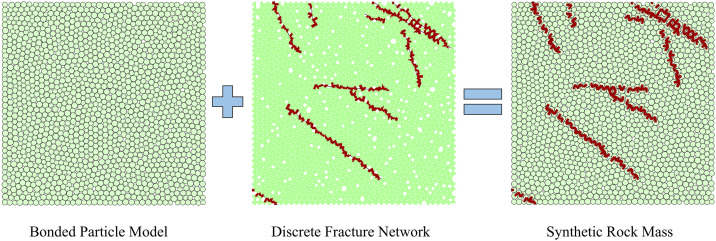
Synthetic rock mass.

The SRM technology [26] represents a method for modeling jointed rock mass, developed on the basis of the discrete element method. In the discrete element software PFC, an intact rock mass is simulated using a bonded particle model (BPM) [33] composed of multiple particles, while the joints within the rock mass are represented by a discrete fracture network (DFN) [34] (Fig 1). BPM is essentially a collection of multiple particles, with interactions between particles achieved by defining various contact models. Forces and torques are generated within the model when relative motion occurs at the particle contact points. If the applied load exceeds the contact strength, contact is disrupted. Consequently, modifying the microscopic contact parameters between particles facilitates the simulation of rock masses with different properties. DFN denotes the aggregate of various fractures within a discrete element model and serves to characterize the size and distribution properties of structural planes within rock masses. The SJCM employed for fractures modifies the interaction between particles, enabling sliding along fracture surfaces between particles on opposite sides [35]. This simulates the mechanical properties of joints in real rock masses. SRM technology employs diverse approaches to simulate rock blocks and fractures, resulting in synthetic rock mass models that closely resemble real-world rock structures. This addresses the limitations of FEM in analyzing jointed rock masses.

### 2.2. Principles of the discrete element model

In this study, PFC^2D^ was employed for numerical simulation experiments. PFC^2D^ is a numerical simulation software grounded in the discrete element method, in which the primary simulation unit is a rigid particle. Particles can undergo translation and rotation, and the interactions between them are simulated through paired contact forces and moments. The macroscopic mechanical behavior of the particle system aligns with the simulated object by specifying properties like the size and dimensions of microscopic particles, as well as the contact parameters between them.

**Fig 2:**
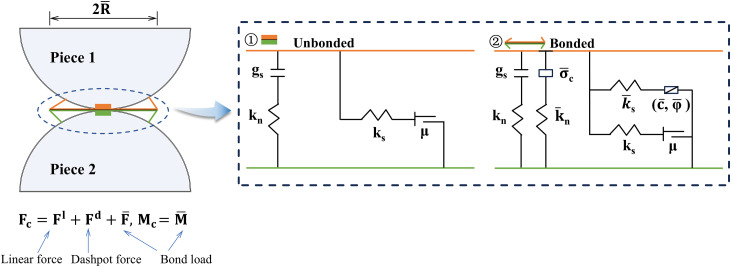
Behavior and rheological components of LPBM.


In the LCC simulation, the linear parallel bond model (LPBM) was selected for the contact between the rock and concrete particles. This model treats particle contact points as springs with normal and shear stiffnesses, which act in parallel with linear component springs. The LPBM exhibits two types of interface behavior (Fig 2): one can be regarded as a linear spring, operating under compression to transmit force only without resisting relative rotation; the other is a parallel bonded spring capable of functioning under both tension and compression conditions, simultaneously transmitting force and moment while resisting relative rotation. This model updates the parallel bonded force and moment according to the force-displacement law in Eq. (1).

$$F_{c}=F^{l}+F^{d}+\overset{―}{F},\;M_{c}=\overset{―}{M}$$ (1)

In the formulas, $F^{l}$ denotes the linear force, $F^{d}$ denotes the dashpot force, $\overset{―}{F}$ denotes the bond force, and $\overset{―}{M}$ denotes the bond moment.

In a two-dimensional scenario, the cross-sectional area (*A*) and moment of inertia (*I*) of the bonds are calculated using Eq. (2), where $R^{\left(\text{1}\right)}$ and $R^{\left(\text{2}\right)}$ denote the radii of the two contacting particles, and λ represents the radius expansion coefficient with a default value of 1.


$$\overset{―}{R}=\overset{―}{\lambda }min\left(R^{\left(\text{1}\right)},R^{\left(\text{2}\right)}\right);\;\overset{―}{A}=\text{2}R;\;\overset{―}{I}=\frac{\text{2}}{\text{3}}{\overset{―}{R}}^{\text{3}}$$ (2)

In the LPBM, when the forces and moments exceed the ultimate strength, the bonds are fractured. The ultimate strength is expressed by Eqs. (3) and (4), respectively.


$$\sigma_{max}=\frac{\overset{―}{F_{n}}}{\overset{―}{A}}+\frac{\overset{―}{R}M}{\overset{―}{I}}$$ (3)

$$\tau_{max}=\frac{\overset{―}{F_{s}}}{\overset{―}{A}}$$ (4)

In the formula, $\sigma_{max}$ denotes ultimate tensile strength, $\tau_{max}$ denotes ultimate shear strength, $\overset{―}{F_{n}}$ denotes normal contact force, $\overset{―}{F_{n}}$ denotes shear contact force, and *M* denotes bending moment.

**Fig 3:**
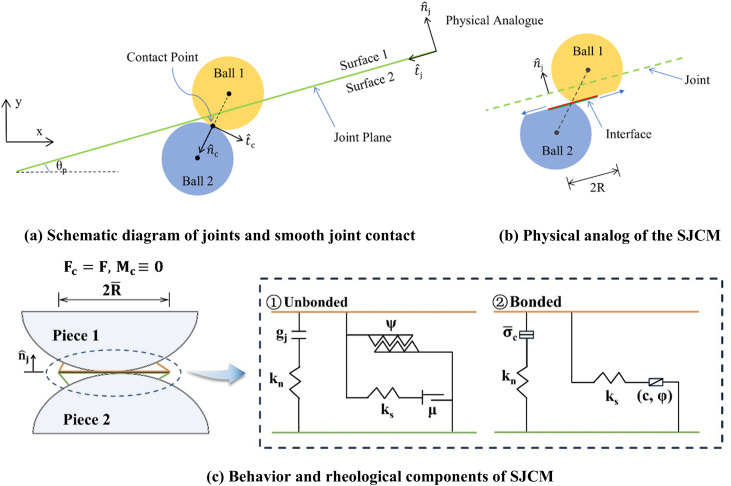
Joint and smooth joint contact model.

A smooth joint model was utilized to replicate the mechanical behavior at the contact interface between limestone and concrete, as well as within the fissure network formed in the limestone. This interface may be either bonded or unbonded, reflecting the particle-to-particle contact dynamics. The mechanical properties of the bonded interfaces demonstrate linear elastic behavior. The unbonded interface forms after bond strength failure and exhibits linear elastic and frictional behaviors, where slip is established by applying Coulomb’s limit to the shear force. The smooth joint model prohibits relative rotation; thus, a pair of particles may overlap or slide rather than move relative to each other, as illustrated in Fig 3(a) and (b)

The force *F* and relative displacement *U* between particles can be expressed using the normal and tangential unit vectors shown in Fig 3(a) as follows: Eqs. (5) and (6), respectively.


$$F=F_{n}\hat{n_{\dot{j}}}+F_{s}$$ (5)

$$U=U_{n}\hat{n_{\dot{j}}}+U_{s}$$ (6)

In these equations, *n* and *s* denote the normal and tangential vectors of the local coordinate system on the joint interface, respectively. Positive values of *F* and *U* represent compression and overlap between the particles. The relationship between force and displacement for smooth joint contact adheres to the Coulomb sliding model with dilation, with the mechanical behavior defined by the normal stiffness $k_{n}$, shear stiffness $k_{s}$, friction coefficient *μ*, cohesion *c*, and dilation angle *ψ*, as shown in Fig 3(c).

The maximum shear force at contact is determined by the interfacial friction coefficient and is calculated using Eq. (7).


$$F_{s}^{\mu }=F_{n}\mu$$ (7)

At the conclusion of each time step, the shear force $\left|F_{s}^{*}\right|$ at the contact point was recalculated. Depending on the relationship between $\left|F_{s}^{*}\right|$ and $F_{s}^{\mu }$, the normal and shear forces at the contact point are updated in two distinct scenarios:

When $\left|F_{s}^{*}\right|\leq F_{s}^{\mu }$ occurs, the normal and shear forces are updated according to Eqs. (8) and (9), respectively:


$$F_{n}:=F_{n}+k_{n}A\Delta \delta_{n}^{e}$$ (8)

$$F_{s}^{*}:=F_{s}-k_{s}A\Delta \delta_{s}^{e}$$ (9)

In the formulas, $\Delta \delta_{n}^{e}$ and $\Delta \delta_{s}^{e}$ represent the elastic components of the normal displacement increment and shear displacement increments, respectively. *A* denotes the cross-sectional area of the smooth joint contact surface. Under two-dimensional conditions, the calculation follows the same procedure as in Eq. (2).

When $\left|F_{s}^{*}\right|>F_{s}^{\mu }$ occurs, it indicates that sliding occurs in the contact, and the normal shear forces are updated according to Eqs. (10)and (11), respectively:


$$\left|F_{s}\right|=F_{s}^{\mu }$$ (10)

$$F_{n}:=F_{n}+\Delta \delta_{s}^{*}tan\;\psi k_{n}A=F_{n}+(\frac{|F_{s}^{*}|-F_{s}^{\mu }}{k_{s}})k_{n}tan\;\psi$$ (11)

## 3. Numerical models and experimental plans

### 3.1. Parameter calibration

The LCC modeling involves two materials. The concrete portion was simulated using the BPM. The influence of random jointing must be considered for the limestone portion. In addition to simulating rocks using BPM, a DFN model is required to simulate the effect of random joints. This study conducted uniaxial compression and direct shear tests on LCCs, using the experimental results as a benchmark to calibrate the micro-parameters of the numerical model. In PFC simulations, the trial-and-error method is frequently employed to adjust the microparameters of the limestone-concrete composite model. This involves continuously adjusting the micro-parameters of particles and contacts until the error meets the numerical testing requirements.

**Fig 4:**
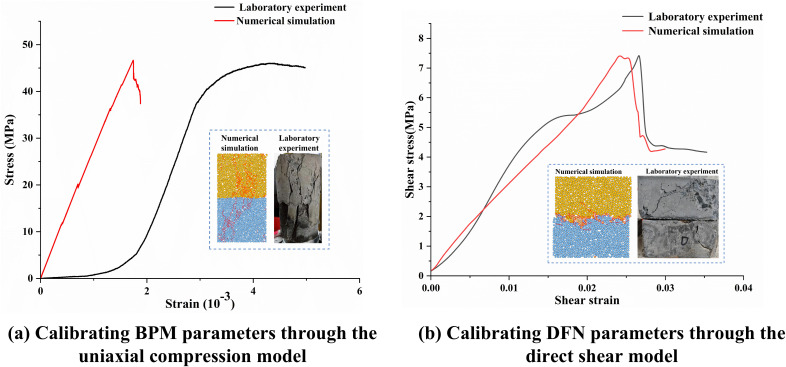
Comparison of calibration parameters for 50 mm diameter model.

The uniaxial compression results of the composite revealed a compressive strength of 46.02 MPa and an elastic modulus of 28.65 GPa, respectively. The microparameters of the BPM were calibrated through uniaxial compression simulations. A trial-and-error approach was employed to calibrate the standard-sized (50 mm × 100 mm) model, ensuring that laboratory tests and numerical simulations yielded identical parameter results and failure modes, as shown in Fig 4(a). This process enabled the determination of the microparameters. To address the computational efficiency issues caused by excessive particle counts, the particle sizes were adjusted based on the model dimensions. Therefore, micro-level parameters and macro-level parameters must be calibrated separately for each size model. The calibration results are presented in Tables 1 and 2.

**Table 1:** Microscopic parameters of particles and linear parallel bond models.

D/mm	R_min_/mm	R_max_/R_min_	Materials	E_c_/GPa	K_n_/k_s_	μ	σ_c_/MPa	τ_c_/MPa	$\overset{―}{{𝐄}_{𝐜}}/𝐆𝐏𝐚$	$\overset{―}{{𝐊}_{n}}/\overset{―}{{𝐊}_{s}}$
50	0.67	1.5	Concrete	48.5	1.1	0.6	31	55	48.5	1.1
			Limestone	50	1.5	0.8	36	100	50	1.5
100	1.30	1.5	Concrete	23	1.1	0.6	25	55	23	1.1
			Limestone	26	1.5	0.8	30	100	26	1.5
200	2.60	1.5	Concrete	19	1.1	0.6	30.5	55	19	1.1
			Limestone	22	1.5	0.8	35.5	100	22	1.5
300	3.90	1.5	Concrete	18	1.1	0.6	26	50	18	1.1
			Limestone	21	1.5	0.8	31	100	21	1.5
400	5.20	1.5	Concrete	18	1.1	0.6	33.5	50	18	1.1
			Limestone	21	1.5	0.8	38.5	100	21	1.5
500	6.50	1.5	Concrete	18	1.1	0.6	30	50	18	1.1
			Limestone	20	1.5	0.8	35	100	20	1.5
600	7.80	1.5	Concrete	18	1.1	0.6	26	50	18	1.1
			Limestone	21	1.5	0.8	31	100	21	1.5
700	9.10	1.5	Concrete	17	1.1	0.6	26	50	17	1.1
			Limestone	20	1.5	0.8	31	100	20	1.5
800	10.40	1.5	Concrete	17	1.1	0.6	27.5	50	17	1.1
			Limestone	20	1.5	0.8	32.5	100	20	1.5

**Table 2:** Macro parameters for models of different sizes.

Model diameter D/mm	Uniaxial compressive strength σ_c_/MPa	Deviation/%	Elastic modulus E/GPa	Deviation/%
50	46.48	1.00	27.95	2.44
100	46.41	0.85	28.14	1.78
200	46.40	0.83	28.67	0.07
300	45.95	0.15	28.14	1.78
400	46.25	0.50	28.54	0.38
500	46.48	1.00	28.59	0.21
600	46.19	0.37	27.84	2.83
700	45.82	0.43	28.19	1.61
800	46.57	1.20	28.57	0.28

Indoor direct shear tests on LCC cube specimens were conducted as the benchmark for calibrating SJCM parameters. The normal stress in the direct shear tests was 3 MPa, with displacement-controlled loading at a rate of 0.1 mm/min. Combining the results from uniaxial compression tests on the LCC, the parameters of the SJCM within the composite model were continuously adjusted. The consistency between laboratory tests and numerical simulations was verified by comparing stress-strain curves and failure modes. As shown in Fig 4(b), the shear strength discrepancy between the two methods was 0.22%. The primary failure zones and fracture propagation paths in the numerical simulation aligned with the laboratory test results. The micro-parameters of the SJCM for the composite material were ultimately determined as presented in the Table 3.

**Table 3:** Microscopic parameters of SJCM.

Parameter type	Normal stiffness k_n_/GPa	Shear stiffness k_s_/GPa	Cohesion c/MPa	Tension σc/MPa	Friction coefficient *µ*
Joint	20	20	0	0	0.8
Interface	100	100	10	10	0.8

### 3.2. Establishment of the composite model

**Fig 5:**
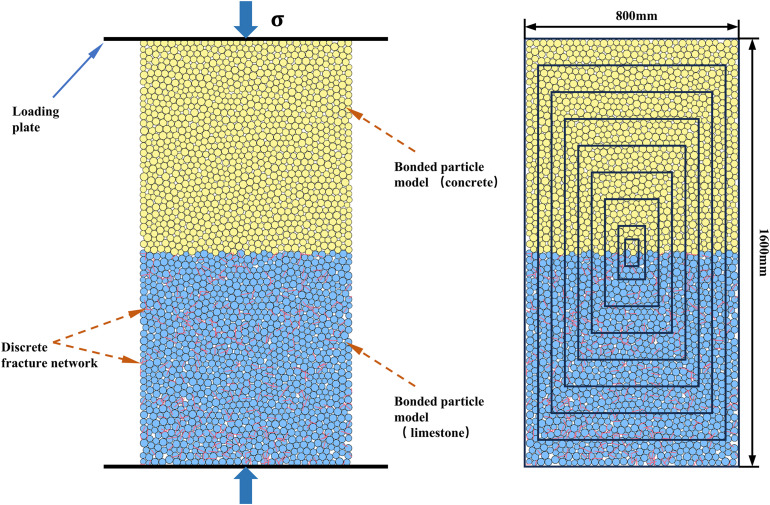
Schematic diagram of LCC particle flow model.

**Fig 6:**
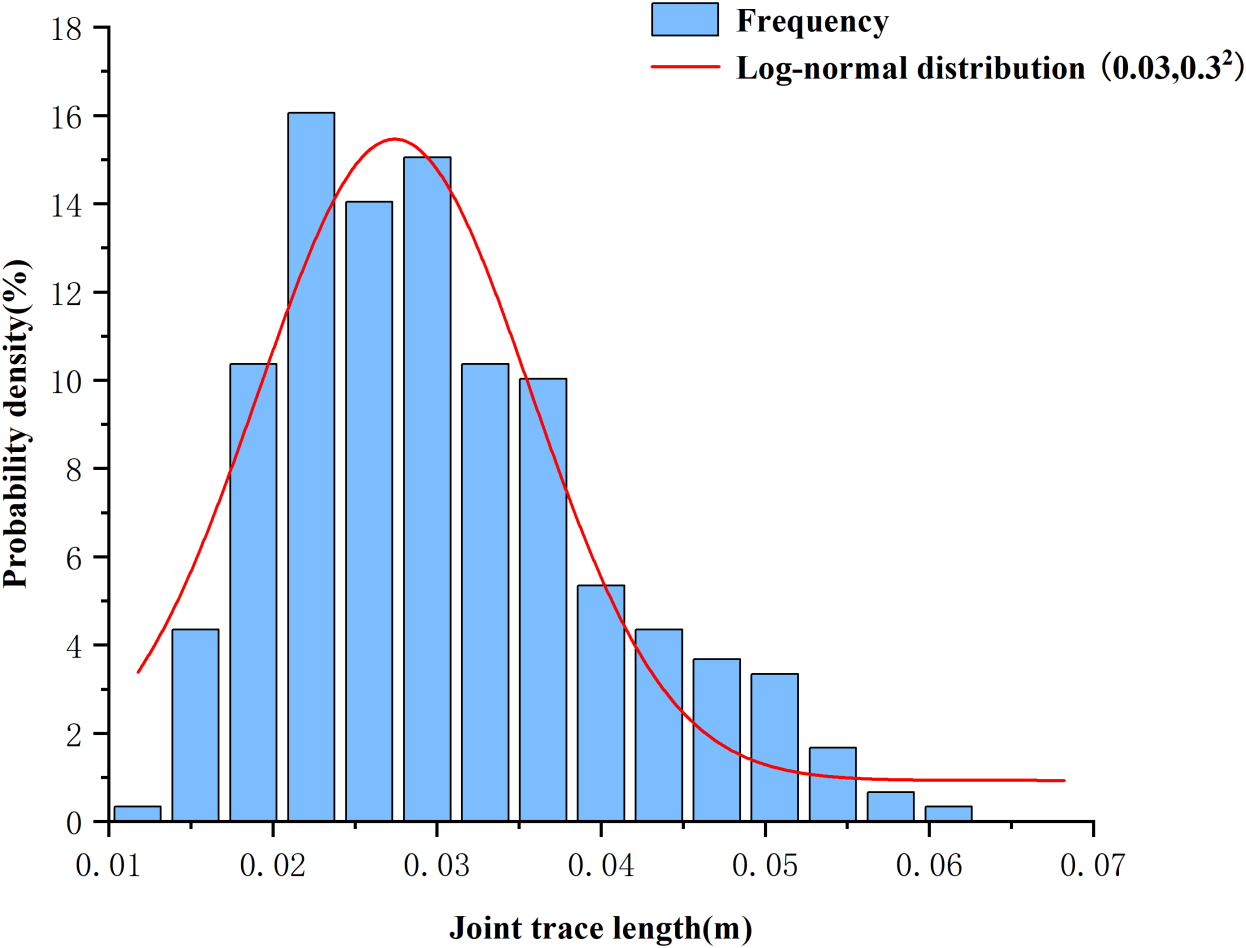
Fracture length distribution histogram.

**Fig 7:**
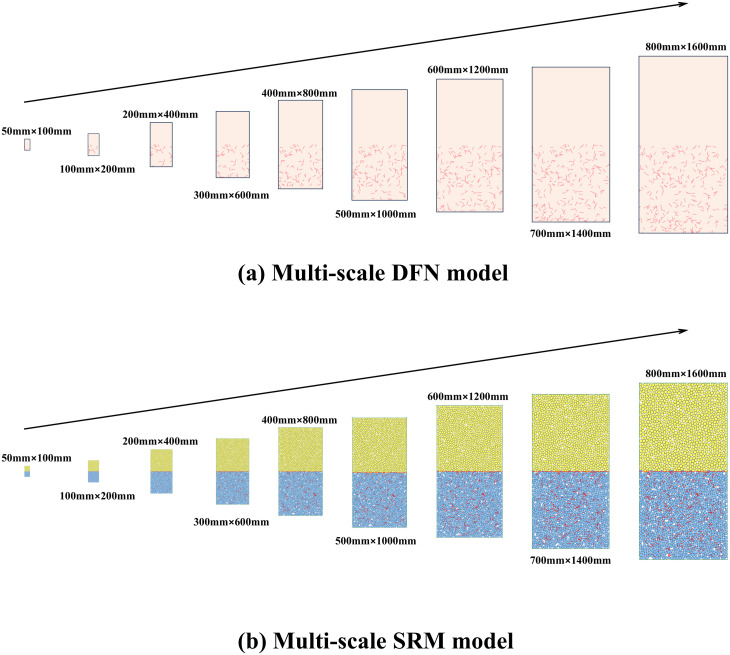
DFN and SRM models of composite for uniaxial compression.

After completing the parameter calibration for the BPM and DFN models, a composite model was established. The upper half of the model represents the BPM for concrete, whereas the lower half comprises the SRM model combining the BPM and DFN, simulating limestone with randomly distributed joints, as shown in Fig 5. The establishment of the composite model involved two steps. First, random fractures were generated within a 1000 × 2000 mm area with a density p of 15 m/m². Both the fracture locations and orientations follow a uniform distribution, whereas the fracture lengths follow a log-normal distribution. Specific distribution parameters are listed in Table 4. Based on these parameters, a frequency histogram of fracture length distribution and a fitted probability density curve are plotted as shown in Fig 6.Within this region, a network of cracks was extracted based on the principle of identical center points but varying dimensions (Fig 5 (b)), yielding DFN models of nine sizes: 50 mm × 100 mm, 100 mm × 200 mm, 200 mm × 400 mm, 300 mm × 600 mm, 400 mm × 800 mm, 500 mm × 1000 mm, 600 mm × 1200 mm, 700 mm × 1400 mm, and 800 mm × 1600 mm. After obtaining the DFN models, they were embedded into BPM models of different sizes that had undergone parameter calibration to generate composite SRM models for the uniaxial compression simulation, as shown in Fig 7.

**Table 4:** DFN distribution characteristics.

Joint characteristics	Distribution model	Distribution characteristics	
Joint length	Log-normal distribution	Mean value/m	Standard deviation/m
		0.0316	0.0097
Joint inclination	Uniform distribution	In different dip angle ranges	
Joint position		In the limestone model area	
Joint density	Constant	15m/m^2^	

It should be noted that, according to the above truncation principle, the model boundary will have a truncation effect on the DFN. For large-size models, the truncated and deleted cracks account for a small portion of the total, and the impact of the “truncation effect” on the model results is within an acceptable range. However, for the 50 mm × 100 mm model, no complete cracks can be truncated, and it is still a complete composite model. In this study, it is regarded as a reference state without fractures to measure the overall degree of decrease in strength and deformation characteristics.

In the simulations of uniaxial compression, the load was applied using displacement control at a speed of 0.01 m/s. During the PFC software loading process, the time step length was set to 4.8 × 10^−8^ s/step. Thus, 0.01 m/s converts to 4.8 × 10^−10^ m/step. Moving the loading plate 1 mm requires approximately 2,083,333 steps. Consequently, the 0.01 m/s rate is sufficiently low, and subsequent analyses of strength and deformation are conducted under quasi-static loading conditions. The calculation precision is set to 1e-5. The simulation terminates when the load decreases to 50% of its peak value. To investigate how the angle of joint inclination affects the size effect in the composite structure, based on the established model above and maintaining consistent parameters, joint dip angle intervals of 0° ~ 5°, 15° ~ 20°, 30° ~ 35°, 45° ~ 50°, 60° ~ 65°, 75° ~ 80°, and 90° ~ 95°. The mechanical properties and the patterns of deformation and failure in these models were analyzed. Owing to space constraints, detailed numerical models for different joint inclinations are not presented here.

Based on the aforementioned modeling, three separate fracture models were created for each size condition at various dip angles using different random seeds (the number of random seeds was set at 10,000, 10001, and 10002, respectively) in order to account for the impact of the DFN randomness on the discreteness of the numerical results. Simulation calculations were then conducted independently. To make sure that the examination of size effects has statistical significance, macroscopic mechanical parameters such peak strength, compressive modulus, and peak strain were statistically examined based on the three sets of simulation data. This paper’s composite model configuration includes changes to the number of random seeds, joint dip angle, and model size. To intuitively demonstrate the control relationships of relevant model parameters in different experimental groups, a parameter hierarchy table was compiled for relevant parameters such as particle size, BPM parameter, SJCM parameter, and DFN parameter, as shown in Table 5.

**Table 5:** Parameter hierarchy description.

Parameter category	Specific parameters	In different specimen size	In different joint inclination	In different random seed	Remarks
Particle size distribution	R_min_, R_max_/R_min_	Varied	Constant	Constant	Adjust particle size distribution according to model size
BPM parameters	E_c_, k_n_/k_s_, σ_c_, τ_c_, μ	Varied	Constant	Constant	Models of different sizes are calibrated separately.
SJCM parameters	k_n_, k_s_, σ_c_, c, μ	Constant	Constant	Constant	The interface and joint parameters remain unchanged.
DFN statistics	Orientation distribution	Constant	Varied	Constant	Controlled inclination angles
	Density, length distribution	Constant	Constant	Constant	Joint statistical parameters remain consistent
DFN realization	Random seed	Constant	Constant	Varied	Used to characterize the randomness of DFN
Boundary/loading	Loading rate, boundary condition	Constant	Constant	Constant	Quasi-static condition

## 4. Numerical results and analysis

### 4.1. DFN randomness analysis and fundamental size effects of LCC

**Fig 8:**
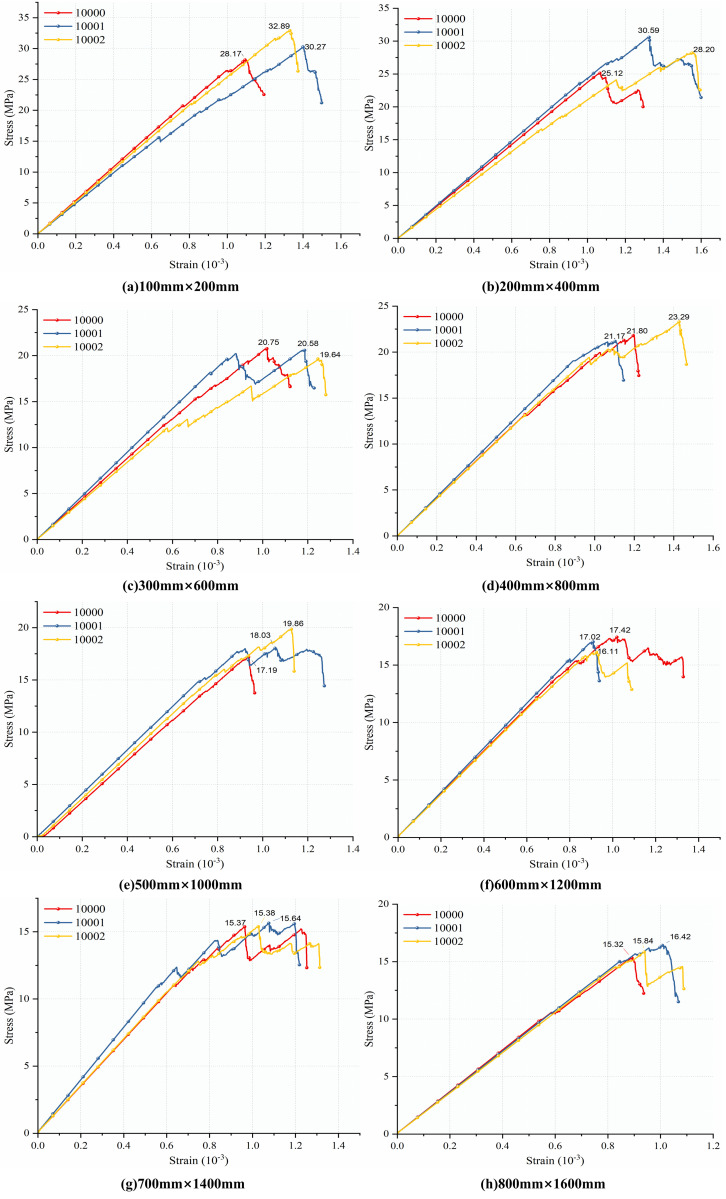
Stress-strain curves of the model under different DFN generation conditions.

As introduced in the previous section 3.2 on the modeling process, uniaxial compression simulations were first conducted on a standard-sized model (50 mm × 100 mm). Subsequently, uniaxial compression tests were performed on multi-scale SRM composite models incorporating random-angle joints at different orientations. This yielded the UCS and compressive modulus for the models of varying scales. This research introduces distinct DFN random seeds for repeated simulations to compensate for the possible randomness of results from a single numerical model under the same working conditions. Fig 8 displays the stress-strain curves for models of various sizes under various DFN random seeds. The stress-strain curves are only shown up to 80% of the peak stress in the post-peak stage for presentational clarity. This truncation merely affects the presentation of the curve; it has no bearing on the study of failure characteristics and damage evolution that follows.

Under various DFN random seeds, the eight composite models of varying sizes display comparable mechanical responses, as illustrated in Fig 8, with the stress-strain curves displaying a steady general trend. Although there are some differences in the peak coordinates and elastic stage slopes of the curves under different DFN random seeds, their fluctuation range is relatively limited. This difference mainly reflects the influence of DFN randomness and also indicates that the results meet the basic requirements for using stress-strain curves for qualitative analysis of size effects. Subsequent quantitative comparisons of mechanical parameters will be based on statistical results, rather than relying on a single curve.

**Fig 9:**
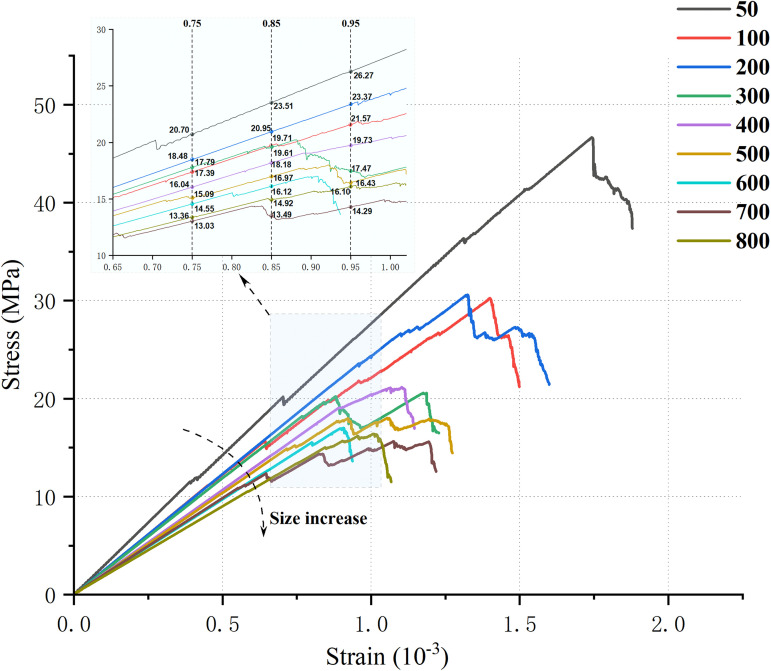
Stress-strain curves of composite models with different dimensions under randomly angled joints.

This study chooses the curve of the test group with 10001 random seeds as the representative result for display because of the consistent stress-strain curve trend of composite models under various DFN random seeds, the representativeness of failure characteristics, and the proximity of mechanical parameters to statistical means. This allows for an intuitive comparison of the mechanical differences of composites of different sizes. The stress-strain curves of composite models of various sizes under uniaxial compression at random dip angles are displayed in Fig 9. As can be seen from the figure, the LCC exhibits obvious size effect characteristics under the action of random dip angle joints: as the size of the composite model increases, its UCS, compressive modulus, peak strain, and other mechanical parameters all show a gradual decreasing trend. This pattern is consistent with the size effect commonly observed in brittle materials such as rock and concrete.

### 4.2. Establishment and fitting of the compressive strength size effect model

In the study of size effects in heterogeneous materials, determining the parameter’s stable scale essentially constitutes an engineering statistics problem rather than a mathematical limit calculation. Macro-mechanical parameters exhibit a gradually converging yet difficult-to-achieve-complete-stability pattern as scale increases. [36–38] Consequently, related studies typically introduce engineering tolerance limits as stability criteria, using the rate of change in macroscopic mechanical parameters between adjacent scales as the basis for judgment. When this rate of change falls below a certain threshold, the corresponding scale is deemed to have achieved statistical representativeness, and further parameter variations are considered unlikely to significantly impact engineering analysis. [39–41] Based on this approach, this study employs a rate of change threshold of less than 10% for adjacent-scale mechanical parameters as the stability criterion to determine the stable scale range for LCC mechanics. This threshold selection comprehensively considers both the statistical fluctuations introduced by DFN and the precision requirements for geotechnical engineering parameters, ensuring both rationality and practicality in engineering-scale analysis.

This section employs the absolute difference ratio to measure the variation in the mechanical characteristics between adjacent dimensional models [40]. The calculation method for the absolute difference ratio *d*_*i*_ is expressed by Eq. (12).


$$d_{i}=\left|\frac{P_{i}-P_{i-\text{1}}}{P_{i-\text{1}}}\right|\times \text{1}\text{0}\text{0}\%$$ (12)

In the formula, $P_{i}$ donates the mechanical parameter of model *i*, and $P_{i-\text{1}}$ donates the mechanical parameter of model *i-1*. The difference ratio allows for an intuitive observation of the trend and magnitude of changes in the mechanical parameters of the models as the dimensions vary. This serves as a quantitative criterion for determining when the relevant parameters stabilize. Typically, a threshold of 10% is used for stability. If the absolute value of the difference ratio for a given mechanical parameter remains consistently below 10% starting from model *i*, it indicates that the mechanical parameter has achieved stability.

**Fig 10:**
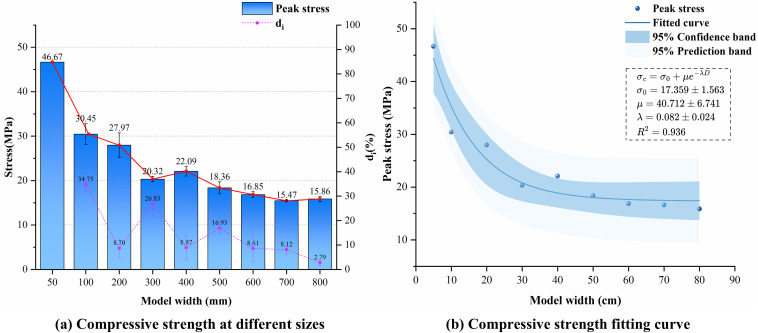
Strength characteristics of random joint dip composites.

Fig 10 illustrates the mechanical behavior of composite models with varying dimensions under uniaxial compression simulations after embedding joints of identical density at random dip angles. Fig 10 (a) shows the mean and standard deviation of the UCS and the$\;d_{i}$ of the composite model under different random seeds as a function of size. The figure demonstrates that the UCS of the composite exhibits a significant decreasing trend as the size increases, while the rate of decrease gradually diminishes, leading to a gradual stabilization of the UCS. This behavior is consistent with the size effect observed in the UCS of rocks. Based on the numerical values of $d_{i}$, the UCS exhibits a significant decrease at model widths of 100 mm, 300 mm, and 500 mm, showing a step-like decline with two distinct scales. The *d* value becomes less than 10% starting from *d*_*600*_, indicating that the UCS of the composite begins to stabilize after the size exceeds 600 mm × 1200 mm.

Nonlinear fitting was performed using the mean and standard deviation of UCS for LCC across different sizes, with results shown in Fig 10(b). The curve demonstrates an exponential decay trend of UCS with increasing size. The confidence band and prediction band in the figure represent the uncertainty of the average size effect trend and the range of strength fluctuations caused by random cracks, respectively. Correspondingly, the legend provides estimated values and fluctuation ranges for different fitting parameters. The specific fitting formula is:


$$\sigma_{c}=\text{1}\text{7}.\text{3}\text{6}\;+\text{4}\text{0}.\text{7}\text{1}{\text{e}}^{-\text{0}.\text{08D}},{\text{R}}^{\text{2}}=\text{0}.\text{9}\text{4}$$ (13)

The fitted curve aligns with the empirical formula for the size effect on rock UCS as proposed by Liu et al. [42], as shown in Eq. (14):


$$\sigma_{c}=\sigma_{\text{0}}+\mu e^{-\lambda D}$$ (14)

In the formula, $\sigma_{c}$ denotes the UCS of a rock specimen with a cross-sectional edge length (or diameter) equal to *D*. The three undetermined parameters $\sigma_{\text{0}}$, μ, and λ depend on the rock properties and its internal natural defect state. $\sigma_{\text{0}}$ can be regarded as the UCS of the rock mass, μ represents the strength difference between the undefected parent rock and the engineered rock mass, and λ is termed the strength decay coefficient, demonstrating the extent to which the strength of the specimen diminishes as its size increases.

### 4.3. Comparative analysis of size effect on deformation properties and strength properties

**Fig 11:**
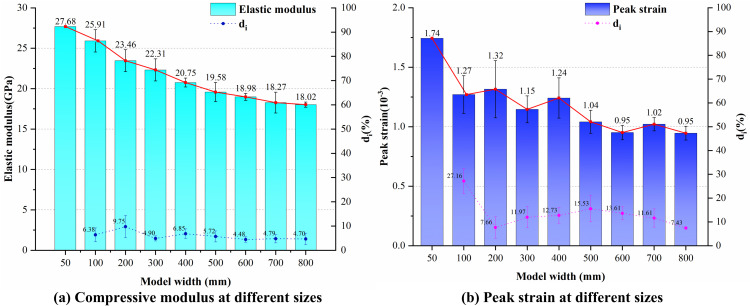
Variation patterns of composite mechanical parameters under randomly inclined joints.

Fig 11 illustrates the variation in the mean and standard deviation of compressive modulus and peak strain with size during uniaxial compression of the LCCs. The dashed lines represent the mean and standard deviation distribution of the ratio of differences for both mechanical parameters. Overall, both the compressive modulus and peak strain exhibit a gradual decrease and stabilization trend with increasing dimensions, similar to the variation pattern of UCS. This indicates that the deformation characteristics of the composite also exhibit a size effect. Regarding the adjacent size difference ratios, the maximum difference ratios for compressive modulus and peak strain were 9.75% and 27.16%, respectively, both lower than the 34.75% observed for UCS. Their average difference ratios were also lower than those of strength parameters, indicating that the deformation characteristics of LCCs exhibit overall weaker sensitivity to size changes compared to their strength properties.

Further analysis reveals that the ratio of compressive modulus differences consistently remains below 10% across the entire size range, whereas the ratio of peak strain differences fails to converge stably below 10%. In Section 4.2, UCS gradually decreases with increasing size, indicating that composite strength follows the weakest-link theory and exhibits typical Weibull statistical size effects. This variation is primarily governed by the statistical distribution of internal defects. As size increases, the probability of crack occurrence and unfavorable crack combinations rises, leading strength to be progressively dominated by defect extremes. Strength stabilizes after the model diameter exceeds 600 mm.

In contrast, the compressive modulus reflects the equivalent average stiffness of the composite structure as a whole. Its value is jointly determined by both the limestone and concrete components and is insensitive to local defects. Furthermore, the volume fraction of cracks and their statistical distribution remain consistent across models of different sizes, resulting in similar initial stiffness fields for each model. This leads to the compressive modulus exhibiting low sensitivity to size variations. Peak strain, however, is highly dependent on local failure path characteristics. Under varying random crack conditions, uncertainties exist in crack propagation patterns, interface slip modes, and primary failure zones, making it difficult to consistently control the adjacent size difference ratio within 10%. The variation in the standard deviation of the difference ratio in Fig 11(b) further illustrates the statistical fluctuation of peak strain under different random seed conditions.

## 5. Discussion

### 5.1. Analysis of size effect in fracture mechanisms based on bond-break and damage maps

**Fig 12:**
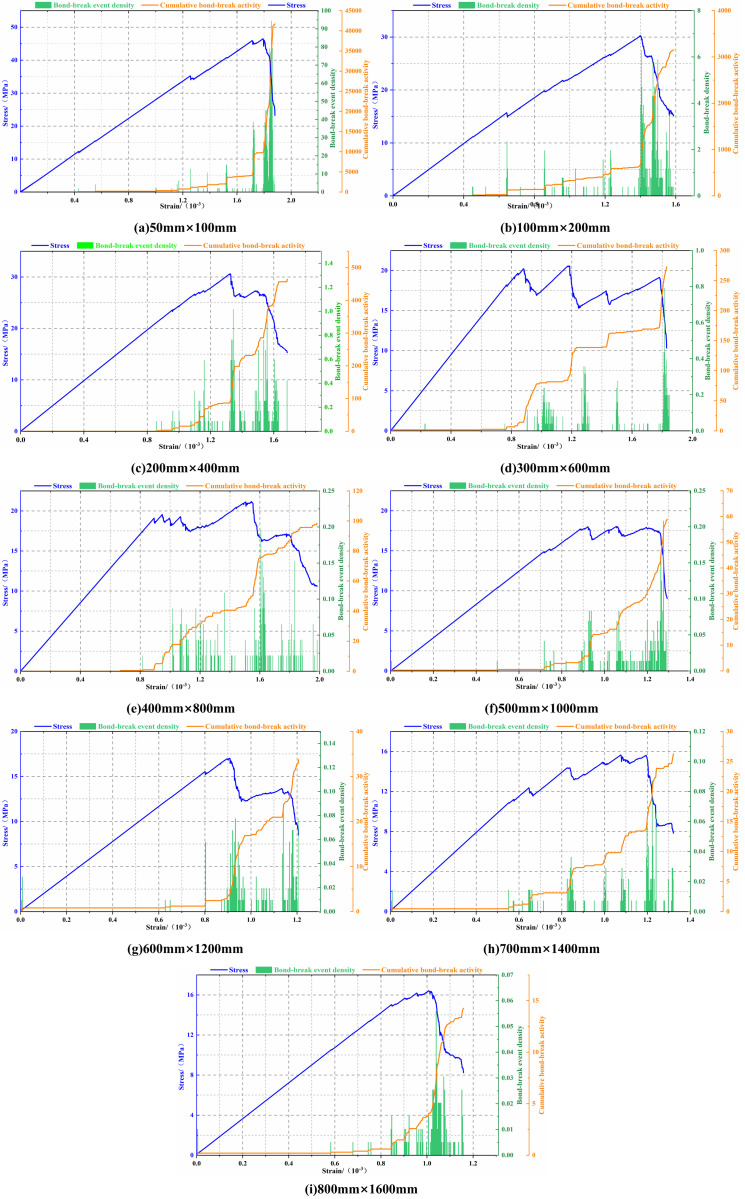
Stress-strain-bond break evolution curves of LCC at different sizes. Note: The bond-break event density in the figure is in units of events/mm²/10 ⁻ ³ strain, and the cumulative bond-break activity represents the cumulative bond-break event density curve.

In uniaxial compression tests, the essence of acoustic emission (AE) phenomena lies in the generation and propagation of internal cracks within rocks under loading conditions, which alter their energy state and release energy in the form of elastic waves. Different stages of uniaxial compression typically exhibit distinct acoustic emission characteristics. In PFC, the generation and cumulative number of cracks are monitored and quantified through changes in the mechanical state of inter-particle contacts. Bond breakage within the contact model can be approximated as the AE signal observed during uniaxial compression simulation. During loading of LCC models of varying dimensions, the number of bond-break events was statistically recorded per unit strain increment to characterize the evolution of micro-damage. To eliminate the influence of model size differences on statistical results and enable comparability across models, the number of bond-breaks per unit strain increment was normalized by the model’s area. The resulting parameter was defined as bond-break event density. Based on this approach, stress, strain, bond-break density, and their cumulative values were recorded during loading for each model. These data were used to plot stress-strain-bond break evolution curves for LCCs of varying sizes, as shown in Fig 12. It should be noted that the bond-break events employed herein serve solely as a qualitative indicator of micro-damage activity. Their evolution primarily aids in analyzing damage characteristics during loading and does not correspond to physically meaningful acoustic emission events.

As shown in Fig 12, the stress-strain-bond break evolution curves of composites of different sizes exhibit relatively consistent characteristics. With increasing strain, the stress in the composites all show a pattern of first increasing and then decreasing, reflecting the typical peak failure characteristics under uniaxial compression. Bond-break events are mainly concentrated in the later stages of loading. With increasing strain, the cumulative bond-break activity curve shows a step-like upward trend, and exhibits a sudden increase when the stress drops sharply, indicating that concentrated failure occurs in the internal contacts of the composite at this stage, and the composite changes from a stable load-bearing state to an overall unstable failure state, with the bond-break event density reaching its peak. The maximum bond-break event density and its cumulative peak count for composites of different sizes during the loading process are summarized in Table 6, used to compare and analyze the differences in the intensity of microscopic damage activity during the failure stage of different sized models.

**Table 6:** Statistical analysis of bond-break parameters for composite models at different sizes.

Specimen width(mm)	50	100	200	300	400	500	600	700	800
Maximum bond-break event density	94.35	6.28	1.02	0.79	0.20	0.21	0.09	0.09	0.06
Peak Cumulative Count	41738.16	3151.80	464.80	272.94	98.46	58.91	33.71	26.26	14.26

According to Table 6, the maximum bond-break event density and cumulative peak count of the composite model generally decrease with increasing size, indicating that size changes have a significant impact on the microscopic damage of the composite. In Fig 12, starting with the model with dimensions of 300 mm × 600 mm, the stress-strain curve of the composite is no longer smooth, and multiple “peaks” appear. This indicates that an increase in dimensions leads to a greater number of internal defects within the composite, heightened structural inhomogeneity, and stress redistribution during loading. Consequently, the failure mode of the composite shifted from a single overall instability to multiple, localized failures. This transition also explains the stepped appearance of the cumulative bond-break activity curve. From an energy perspective, larger composite models contain more internal defects but with a more uniform distribution. During fracture propagation, crack initiation and growth become more dispersed, thereby diluting the peak energy release. This reduces the occurrence of large-scale, violent energy release events, and the material failure tends toward stabilization, avoiding the high peak and short duration of bond-break event density observed in smaller specimens (e.g., 50 mm × 100 mm) during failure. This also explains the gradual decrease in both the maximum bond-break event density and peak cumulative count as the specimen size increased.

**Fig 13:**
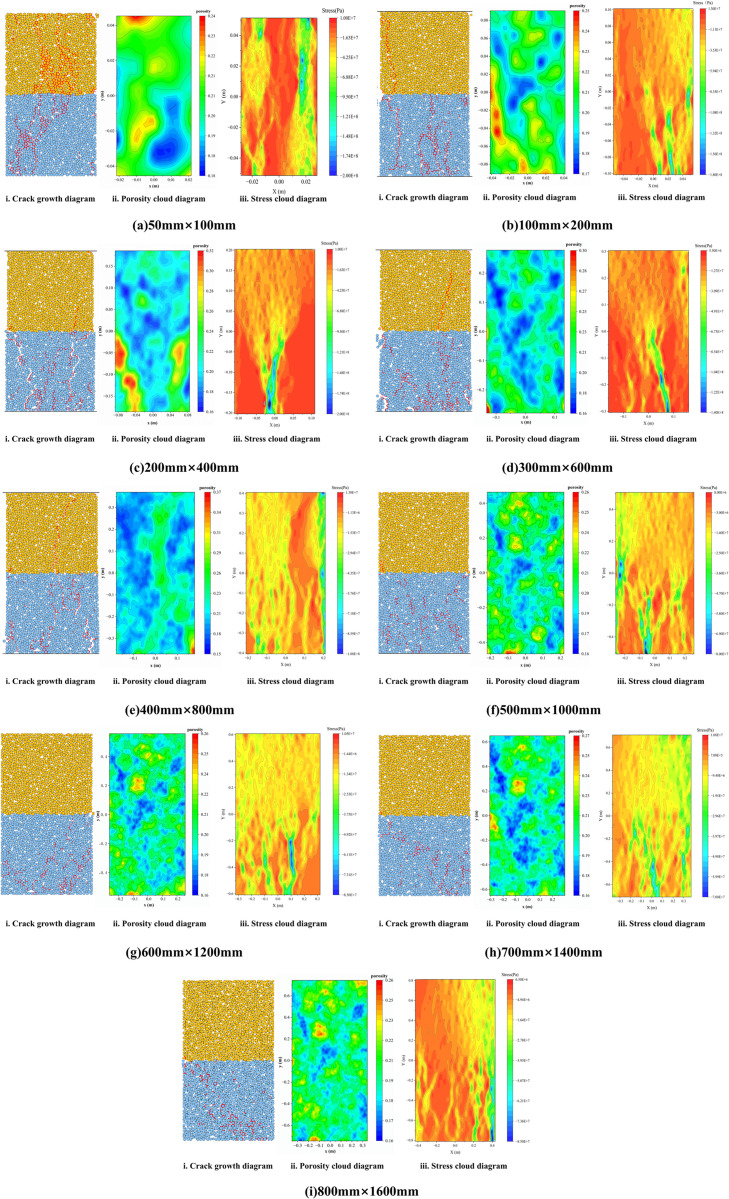
Crack growth diagrams, porosity cloud diagrams, and stress cloud diagrams for composites at different sizes.

In PFC^2D^, porosity characterizes the density of local particle distribution. Following loading failure, regions with higher porosity typically correspond to more concentrated micro-damage and failure. Based on this principle, measurement circles covering the entire model area were arranged in LCC models of varying sizes, maintaining consistent statistical resolution. By extracting the coordinates and porosity data from each measurement circle, a porosity cloud map of the composite after failure was generated. Simultaneously, by exporting the coordinates and contact force information of different particles, a stress distribution map (y-direction) is plotted for the composite after failure. Integrating the crack distribution map, porosity cloud map, and stress cloud map of the composite after failure, as shown in Fig 13, the influence of size variation on the failure mode and damage evolution of the LCC is investigated.

Fig 13 illustrates the failure characteristics of LCCs of varying sizes from the perspectives of crack initiation and propagation, pore distribution, and stress distribution. In terms of crack propagation, as the size of the composite increases, the number of cracks tends to decrease, and the paths they take become progressively shorter. When the model dimensions are small, cracks propagation traverse the contact interface, resulting in the overall failure of both concrete and limestone. Once the composite dimensions reach 500 mm × 1000 mm, the increasing number of joints progressively weakens the limestone. The failure of the limestone portion becomes dominant, leading to a decrease in the number of cracks during composite failure. Furthermore, the propagation path cannot traverse the contact interface to enter the concrete.

The planar distribution characteristics of porosity and stress contour maps show good consistency with crack propagation paths. In areas where cracks are concentrated, porosity increases significantly, corresponding to locations of significant damage and failure within the composite. Simultaneously, these areas typically represent stress concentration points in the stress contour maps. This correspondence indicates that these regions play a major role in stress transfer during loading, with localized stress concentrations promoting crack initiation and propagation, ultimately forming the main failure zone. The stress contour maps show that when the model size is less than 500 mm × 1000 mm, a relatively clear and complete stress transfer path exists within the LCC, with a relatively simple force chain structure. As the size increases further, the stress transfer path within the composite gradually exhibits a network-like characteristic, with force chains often appearing as a mesh structure formed by interwoven incomplete short chains. This variation pattern is consistent with the “joint weakening effect” described earlier. As the size increases, the number of internal defects in the composite increases, the non-uniformity gradually increases, and the stress is more likely to be interrupted when it is transmitted to local weak areas. It can only continue to be transmitted by redistributing the path, thus forming the characteristics of an increase in the number of stress paths, a decrease in the width of a single path, and their interweaving.

### 5.2. The regulatory role of joint dip intervals in size effect rules

According to the relevant introduction on composite model establishment in Section 3.2, theoretically, 72 distinct composite numerical models can be constructed across 9 size categories and 8 joint dip angle ranges (including random dip angles). However, due to the wide span of model sizes, the mean joint length is uniformly set to 0.03 m to ensure the rationality of fracture distribution. Under these conditions, numerical models with standard dimensions of 50 mm × 100 mm cannot capture complete fractures during DFN extraction. Therefore, this size model represents a complete complex with no variation in joint dip angle intervals. This standard-size model serves as the fracture-free baseline state for longitudinal comparative analysis within the same test group. Based on the above considerations, a total of 65 distinct numerical complex models can ultimately be obtained.

**Fig 14:**
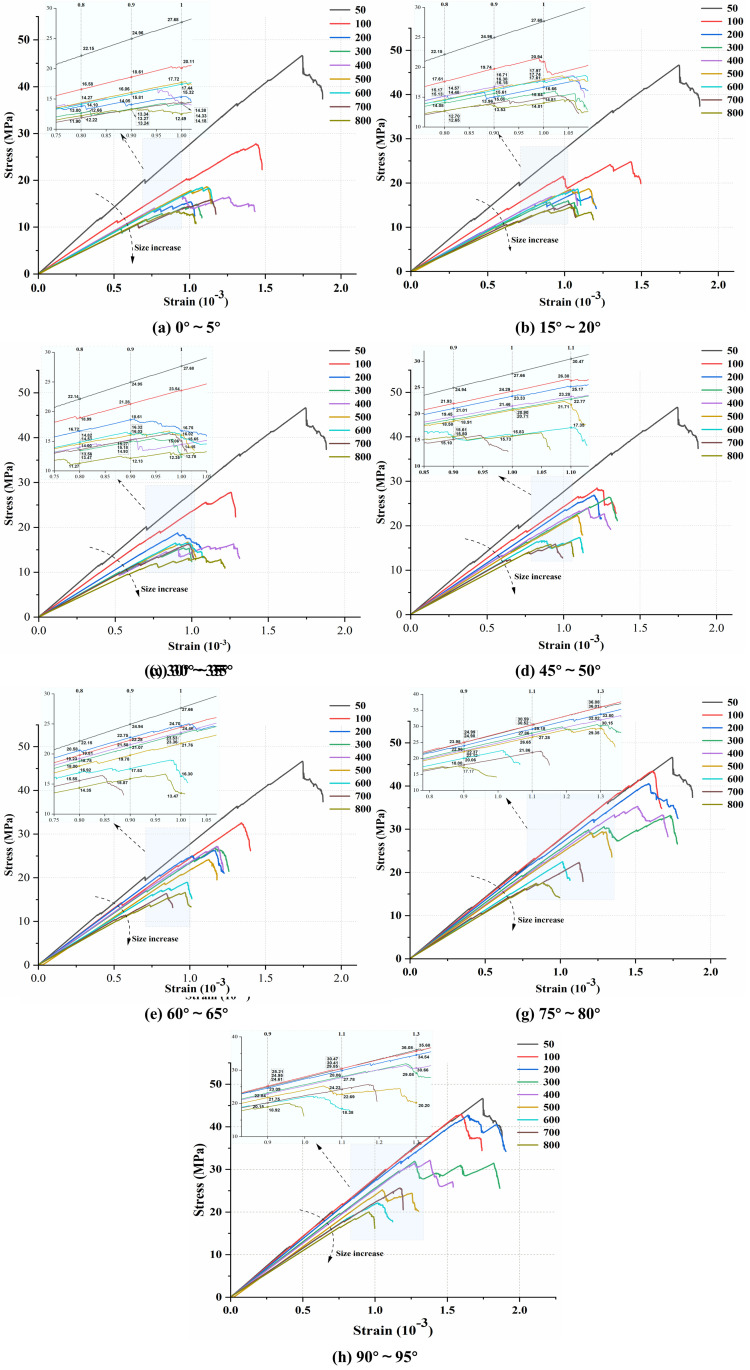
Uniaxial stress-strain curves of composite models at different scales under joint action at various dip angle ranges.

This section selected 7 joint dip angle ranges (excluding the random dip angle group), totaling 56 fractured models for uniaxial compression numerical simulation. Based on this, the DFN generation process was repeated three times by altering the random seed number to account for the influence of fracture randomness on size effects. By comparing simulation results under different conditions, the impact of varying joint dip angle ranges on the size effects of complex strength and deformation characteristics was investigated. Based on the uniaxial compression numerical simulation results, representative random seed test groups are selected for presentation in this section. To ensure the readability of stress-strain curves and overall presentation effectiveness, the post-peak stages of stress-strain curves for different models are uniformly truncated at 80% of peak stress, as shown in Fig 14.

**Fig 15:**
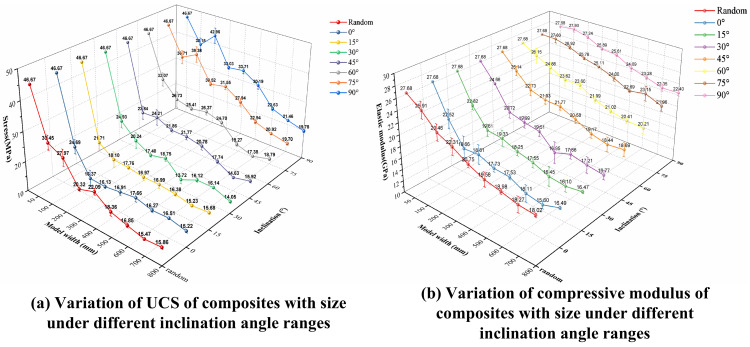
The effect of joint dip angle range on the size effect of composite strength and deformation characteristics.

As shown in Fig 14, under different joint dip angle ranges, the peak value and slope of the stress-strain curve of the LCCs decrease and tend to stabilize with increasing size, reflecting the size effect characteristics of the composite in deformation and strength response. Meanwhile, the decreasing trends of the peak value and slope of the stress-strain curve with increasing size differ under different dip angle ranges, indicating that the change in fracture dip angle has a certain impact on the size effect on strength and deformation characteristics. To further quantitatively analyze the differences in size effect under different dip angle ranges, peak strength and elastic modulus were extracted from the stress-strain curves of all models, and the relationship between the mean values of these two mechanical parameters and size under different dip angles was plotted, as shown in Fig 15.

Combining Figs 14 and 15(a), it can be seen that, under the same size conditions, the UCS of the composite generally increases with the increase of the joint dip angle, indicating that the weakening effect of joints on the composite strength gradually decreases with the increase of the dip angle. Taking the random dip angle test group as a reference, when the dip angle is less than 45°, the variation law of the composite UCS with the increase of size is basically consistent with that of the random dip angle group: within the small size range, the strength shows a rapid decreasing trend with the increase of size, among which the largest strength decrease between adjacent sizes occurs in the 15° to 20° test group, and the UCS of the model with a diameter of 100 mm decreases by 53.48% compared with the previous size; when the model diameter exceeds 600 mm, the UCS gradually tends to stabilize. When the joint dip angle reaches 45° and above, the overall UCS of the composite still decreases with the increase of size, but its decrease is significantly smaller than that of the low dip angle test group, and it has not yet shown a significant stabilizing trend within the size range set in this paper. Based on the characteristics of the broken line variation, the UCS of the 90° ~ 95° test group was higher than that of the corresponding size models of other test groups at all sizes, and its overall strength reduction with size change was also the smallest.

Because the overall trend consistently decreased, we used the total reduction *Δσ* to measure the prominence of the size effect on the UCS of the composite across different joint dip angle ranges. A larger *Δσ* indicates a more pronounced size effect in the composite. The formula for calculating *Δσ* is given by Eq. (15) below:


$$\Delta \sigma =\sigma_{\text{1}\text{0}\text{0}}-\sigma_{\text{8}\text{0}\text{0}}$$ (15)

In the formula, $\sigma_{\text{1}\text{0}\text{0}}$ denotes the UCS of the composite model with a width of 100 mm; $\sigma_{\text{8}\text{0}\text{0}}$ denotes the UCS of the composite model with a width of 800 mm.

**Fig 16:**
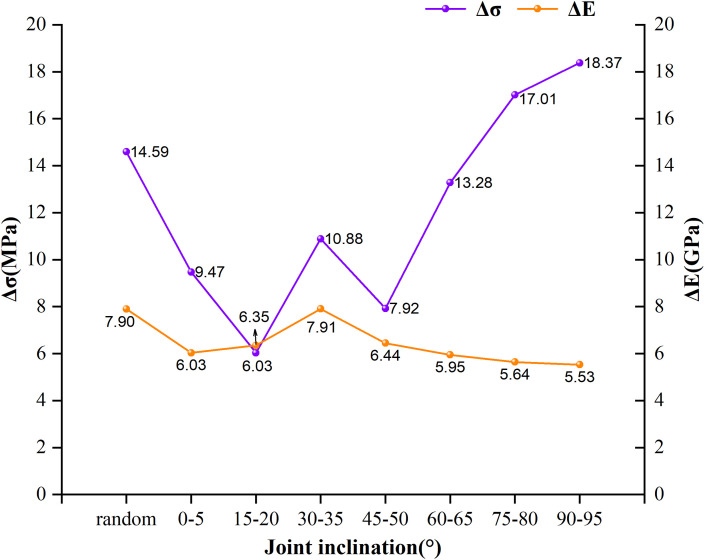
Δσ and ΔE values at different dip angle ranges.

Similar to *Δσ*, we can also use *ΔE* to describe the degree of size effect on the compressive modulus of the composite. Its calculation method is identical to that of *Δσ*. From Fig 15, we extracted the mean UCS and mean compressive modulus of composite models with dimensions of 100 mm × 200 mm and 800 mm × 1600 mm for different joint dip angle ranges. Eight sets of *Δσ* and *ΔE* values were calculated for each dip angle range, summarized in Table 7, and their variation trends across dip angle ranges are visually presented in Fig 16.

**Table 7:** Δσ and ΔE value`s at different dip angle ranges.

Inclination range	Random angle	0° ~ 5°	15° ~ 20°	30° ~ 35°	45° ~ 50°	60° ~ 65°	75° ~ 80°	90° ~ 95°
Δσ/MPa	13.59	14.43	10.24	14.28	12.17	15.92	25.87	22.85
ΔE/Gpa	6.39	4.27	6.34	8.23	7.01	5.04	6.60	6.50

As shown by the broken line of *Δσ* in Fig 16, there are significant differences in the overall reduction of UCS of the LCCs under different joint dip angle ranges. The *Δσ* values corresponding to each dip angle range are approximately distributed between 6.0 and 19.0 MPa, with the smallest reduction occurring in the 15°–20° dip angle range, while the largest reduction corresponds to the 90°–95° dip angle range. This indicates that the influence of joint dip angle on the size effect of composite strength has obvious non-uniformity. Compared with the random dip angle test group, it can be seen that under specific dip angle conditions, the size effect may be further amplified or weakened.

Further analysis revealed that when the joint dip angle exceeded the 45°–50° range, the *Δσ* value showed a significant increasing trend, indicating a substantial enhancement in the size effect of the composite strength. This phenomenon is consistent with the pattern reflected in Fig 15(a), namely, under large dip angle conditions, the UCS of the composite is not easily stabilized within the size range set in this paper. For the low dip angle test group, the strength reduction was mainly concentrated in the small and medium size stage. When the size reached approximately 600 mm × 1200 mm, the strength change tended to level off. However, in the larger dip angle range, the strength reduction process with increasing size continued to extend to a larger scale range, resulting in a significant increase in the overall strength reduction *Δσ*.

Combining the variation characteristics of the stress-strain curve slope in Fig 14 and the broken line trend in Fig 15(b), it can be seen that the compressive modulus of the LCCs exhibits a significant size effect under different joint dip angles, with a generally consistent overall trend, but some differences in the magnitude of reduction. Numerically, the change in the composite’s compressive modulus between adjacent dimensions is relatively uniform, and its overall reduction is significantly smaller than that of the UCS. In terms of the trend, the compressive modulus in different dip angle ranges gradually stabilizes with increasing size, which is consistent with the analysis results of the size effect on the composite’s deformation characteristics in Section 4.3. Further comparison of the broken line plots of the compressive modulus with size under different dip angle ranges reveals that the compressive modulus at different sizes generally increases with increasing joint dip angle, indicating that increasing the joint dip angle weakens the size effect of the composite’s compressive modulus to some extent.

The *ΔE* values in Table 7 and the *ΔE* line in Fig 16 reflect the overall decrease in the compressive modulus of the composite as the size increases under different joint dip angle ranges. The *ΔE* values corresponding to each dip angle range are approximately distributed between 5.5 and 8.0 GPa, with a minimum of 5.53 GPa corresponding to the 90°–95° dip angle range and a maximum of 7.91 GPa corresponding to the 30°–35° dip angle range. Comparing the two lines in Fig 16, it can be seen that the dispersion of *ΔE* is significantly less than that of *Δσ*, and the *ΔE* values for most dip angle ranges are smaller than those for the random dip angle test group. This indicates that compared with UCS, the size effect of the composite compressive modulus is less sensitive to the joint dip angle, and the ordered distribution of joint dip angles tends to weaken the size effect of the compressive modulus.

## 6. Conclusion

This study employs the two-dimensional discrete element software PFC^2D^ to conduct numerical simulations of LCCs. Utilizing SRM to embed random joints within the limestone matrix, multiple sets of uniaxial compression tests were performed on LCCs under different dimensional configurations and joint dip angle ranges. The study focuses on analyzing the size effects of strength and deformation characteristics under uniaxial compression for the composite under random joint inclination conditions. Building upon this, it systematically investigates the influence of varying joint inclination ranges on the scale effects of the composite’s UCS and compressive modulus. The conclusions reveal the mechanical mechanisms linking joint geometry and scale effects under 2D conditions. Their extension to three-dimensional composites requires further validation. Specific findings are as follows.

(1) Under 2D uniaxial compression, the UCS, compressive modulus, and peak strain of LCCs decrease and stabilize as size increases. The UCS enters a stable range after the model size reaches 600 mm × 1200 mm. The compressive modulus decreases slowly, stabilizing with a difference ratio below 10%. Although peak strain decreases overall, it fluctuates significantly due to random crack distribution and fails to stabilize within the tested range. Notably, deformation characteristics are less affected by size effects than strength characteristics at the 2D level.
(2) As size increases, the maximum bond-break event density and cumulative peak count generally decrease. Larger 2D models contain more internal defects, causing crack initiation and propagation to become more dispersed. This shifts the energy release process from concentrated to discrete, significantly weakening peak characteristics. Consequently, the failure mode transitions from overall instability at small sizes to multiple local failures at larger sizes.
(3) With increasing size, the number of failure cracks decreases and propagation paths shorten. In models smaller than 500 mm × 1000 mm, cracks easily penetrate the limestone-concrete interface, leading to overall failure with simple, clear stress transmission paths. In larger models, limestone failure dominates as cracks struggle to penetrate the interface into the concrete. The internal stress transmission evolves into a networked structure, characterized by intertwined, incomplete short-chain force chains.
(4) Across seven joint dip angle ranges, LCCs exhibit distinct size effect characteristics. The weakening effect of joints on UCS decreases as the dip angle range increases. Using the 45° to 50° range as a boundary, groups with smaller dip angles show size effect laws similar to random groups. However, when dip angles exceed this range, UCS fails to show a clear stabilization trend within the tested dimensions. Conversely, the joints’ weakening effect on the compressive modulus decreases with larger dip angles, showing uniform and stable changes across all ranges.
(5) Quantitative comparison using *Δσ* and *ΔE* reveals that UCS size effects are relatively small at low dip angles but intensify significantly above 45° to 50°, peaking in the 90° to 95° range. For the compressive modulus, the size effect is weakest at 90° to 95° and strongest at 30° to 35°, with minor differences elsewhere. Overall, the influence of joint dip angle on UCS size effects involves certain uncertainty and can be amplified under specific conditions. In contrast, the compressive modulus size effect is less sensitive to joint orientation, and orderly dip angle distributions may even weaken its size effect.

## Supporting information

S1 File Data. (ZIP)
